# Integrative effects of morphology, silicification, and light on diatom vertical movements

**DOI:** 10.3389/fpls.2023.1143998

**Published:** 2023-03-27

**Authors:** Alessandra Petrucciani, Paolo Moretti, Maria Grazia Ortore, Alessandra Norici

**Affiliations:** Dipartimento di Scienze della Vita e dell’Ambiente, Università Politecnica delle Marche, Ancona, Italy

**Keywords:** frustule, sinking, dynamic light scattering, diatom, ecophysiology, stable isotopefractionation

## Abstract

Diatoms represent the most abundant and diversified class of primary producers in present oceans; their distinctive trait is the ability to incorporate silicic acid in a silica outer shell called frustule. Numerous adaptative functions are ascribed to frustules, including the control of vertical movements through the water column; this indirectly determines cell access to fundamental resources such as light and nutrients, and favors diatom escape from predators. At the same time, light guides phototroph movements in the water column by affecting cell density (e.g., by modulating Si deposition in diatoms, vacuole volume, and/or solution). We investigated how the tremendous diversity in morphology and silicification that characterizes the frustule and the crucial role of light in diatom spatial distribution govern diatom sinking capacity. To test their integrative effects, we acclimated four diatoms distinguished by frustule traits (*Chaetoceros muelleri*, *Conticribra weissflogii*, *Phaeodactylum tricornutum*, and *Cylindrotheca fusiformis*) to different light conditions and evaluated their physiological performance in terms of growth, elemental composition, morphological changes, and their *in vivo* sinking capacity. What emerged from this study was that silicification, more than other morphological characteristics, controls species vertical movements, while a higher energy availability enhances cell floating independently from the silica content.

## 1 Introduction

Diatoms are a greatly diversified and successful group of eukaryotic phototrophs (Bacillariophyceae) belonging to the supergroup Stramenopiles (Bowler et al., 2010). The ability to incorporate Si in frustules is a dominant feature in diatoms. Although its original role is still controversial, a frustule more than a cellulosic or carbonate cell wall can increase cell density and, therefore, contribute to the faster sinking of diatoms as compared to that of other phytoplanktonic groups (Martin-Jézequel et al., 2000; Raven and Waite, 2004; Marron et al., 2016). Consequently, diatoms become significant players not only in the biological C pump (Jin et al., 2006; Tréguer et al., 2018), but also in Si precipitation to the bottom of the ocean (Moriceau et al., 2007; Sutton et al., 2018).

In nature, frustules are diverse in terms of morphology and silicification (Armbrust, 2009; Malviya et al., 2016; Hildebrand et al., 2018). Several studies support the hypotheses that cell size, shape, and complexity have a crucial role in maintaining a favorable position in the water column (Allen, 1932; Reynolds, 2006; Durante et al., 2019). It is evident that a bigger cell volume leads to an increased sinking rate, and, at similar size, spherical organisms have a higher settling velocity than elongated ones (Mcnown and Malaika, 1950; Smayda, 1970; Smayda and Bienfang, 1983; Durante et al., 2019); furthermore, several morphological adaptive attributes (such as spines) have been developed in response to environmental pressure (Padisak et al., 2003; Sommer et al., 2016). From an ecological and temporal perspective, we can assume that phytoplankton, thus diatoms, has evolved geometrical trade-offs required to thrive in advantageous niches (Durante et al., 2019).

Moreover, frustule traits indirectly control sinking capacity by changing cell density. In particular, Si deposition shows a phenotypical response to different external environmental factors (i.e., predators, nutrient availability, pH, temperature, salinity, and light intensity) affecting cell density (Durbin, 1977; Brzezinski et al., 1990; Flynn and Martin-Jezequel, 2000; Pondaven et al., 2007; Vrieling et al., 2007; Hervé et al., 2012; Shrestha et al., 2012; Su et al., 2018; Xu et al., 2021; Petrucciani et al., 2022a). Among them, light is a crucial driver in the distribution of phytoplankton through the water column, not only affecting their photosynthetic performance, but also influencing cell density. Vacuole dimensions and its solute composition along with the silicification degree are among possible ways to modulate cell density (Lavoie and Raven, 2020; Xu et al., 2021). Nevertheless, direct findings assessing the light role on diatom Si deposition are still controversial: according to some authors, light deficiency enhances Si deposition in frustules (Xu et al., 2021), while other authors suggest that the latter is directly related to the increase in light intensity (Su et al., 2018).

New insights into the fascinating diversity of diatoms also confirmed that very small and scarcely silicified diatoms (*Minidiscus* sp., Leblanc et al., 2018) are able to rapidly sink out: their impact on Si and C exports to the bottom of the oceans is significant even though their small size and biomineralization do not classify them as good sinkers. These new observations open a lot of questions on the relation between the huge diatom diversity in size and silicification, and their contribution to the C and Si cycles (Tréguer et al., 2018).

By altering diatom spatial distribution, sinking/buoyancy affects the cell access to light and nutrients (Smayda, 1970; Margaref, 1978; Falciatore et al., 2000). Among the cellular mechanisms involved in buoyancy control (Waite et al., 1992; Raven and Doblin, 2014; Lavoie et al., 2016; Arrieta et al., 2020; Lavoie and Raven, 2020), new insights reveal the existence of an unsteady sinking behavior in which cells vary the sinking speed over more than an order of magnitude repeatedly within tens of seconds, in response to physiological and environmental conditions (Gemmell et al., 2016; Du Clos et al., 2019; Du Clos et al., 2021). These results evidence the fact that diatoms can take advantage of patchy distributions of nutrients and/or escape from predators by controlling buoyancy over short time scales (Raven and Waite, 2004; Du Clos et al., 2021).

The aim of this work was to investigate how diatom sinking capacity depends on morphological diversity, silicification, and light as factors governing vertical movements: the integrative effects of factors commonly treated in isolation have been addressed. To achieve this purpose, four distinct diatoms in terms of size, shape, and silicification (*Chaetoceros muelleri*, *Conticribra weissflogii*, *Phaeodactylum tricornutum*, and *Cylindrotheca fusiformis*) were acclimated to different light conditions (15, 60, and 180 µmol photons·m**^−^** ^2^ s**^−^** ^1^). Diatom physiological performance in terms of growth, change in morphology, photosynthetic efficiency, and inorganic composition was investigated. In order to directly assess the *in vivo* sinking capacity of diatoms, dynamic light scattering (Berne and Pecora, 1976) was here exploited for the first time to our knowledge. Cell size distribution in solution was also confirmed by means of this physical technique (Andreozzi et al., 2019).

## 2 Materials and methods

### 2.1 Algal cultures

Cultures of morphologically distinct diatom species were established in 250-ml flasks filled with 100 ml of AMCONA medium (Fanesi et al., 2014), and maintained in a culture chamber at 18°C, illuminated with cool white fluorescent lamps (OSRAM Lumilux 36W/840^1^) at 12:12 h light–dark cycles. The centric species selected for the experiments were *C. muelleri* (CCAP 1010/3, https://www.ccap.ac.uk/) and *C. weissflogii* (DCG 0320, https://bccm.belspo.be/about-us/bccm-dcg), while *C. fusiformis* (NEPCC417) and *P. tricornutum* (DCG 0981) were chosen among the raphid pennate species. Diatoms were acclimated for at least 10 generations to three different light intensities (15, 60, and 180 µmol photons·m**^−^** ^2^ s**^−^** ^1^). All measurements were performed during the late exponential phase from batch cultures.

### 2.2 Specific growth rate, cell volume, and dry weight

Cell number and cell volume were measured using a CASY TT cell counter (Innovatis AG, Reutlingen, Germany) as described in Petrucciani et al. (2022b). Specific growth rates, *μ*_max_, were derived from a non-linear regression of the daily measured cell density, carried out on a minimum of three distinct cultures for each treatment. The model used was β-function (Yin et al., 2003), where *N* represents the algal density, *C_m_* is the maximum cell density growth rate in the linear phase, *t_m_* is the inflection point at which the growth rate reaches its maximum, *t_b_* is the reference time for the beginning of the growth process, and *t_e_* is the time at which the growth ends. The best-fitting method for each biological replica was related to the highest coefficient of determination (*r* ^2^).


(1) $$\frac{dN}{dt}=\;C_{m}\left(\frac{t_{e}-t}{t_{e}-t_{m}}\right){\left(\frac{t-t_{b}}{t_{m}-t_{b}}\right)}^{\frac{t_{m}-t_{b}}{t_{e}-t_{m}}}\;$$

(2) $$\mu_{max}=\frac{C_{m}}{N\left(t_{m}\right)}$$

Therefore, *C_m_* value was used to obtain *μ*_max_ following Eq. 2, where *N*(*t_m_*) represents the density of cells achieved at time *t_m_*.

Diatoms collected during the exponential phase were put in pre-weighted tubes and dried at 80°C till a stable cellular dry weight is attained. All measurements were carried out on samples from three distinct cultures.

### 2.3 Quantification of silicon

The cellular content of Si was measured in diatoms collected during the exponential phase using a total reflection x-ray fluorescence spectrometer (S2 Picofox, Bruker AXS Microanalysis GmbH, Berlin, Germany), as reported in Petrucciani et al. (2022b). Spectral deconvolution and quantification of elemental abundances were performed by the SPECTRA 6.1 software (Bruker AXS Microanalysis GmbH, Berlin, Germany).

### 2.4 Sinking capacity

Dynamic light scattering (DLS) measurements were carried out using a Malvern Zetasizer PRO system in backscattering mode, with temperature controlled at 25°C. All studies were performed at a 173° scattering angle, and short time measurements were carried out, with consecutive measurements for each sample. The diatom solution system is illuminated by laser light, and the scattered radiation is detected as a function of time. Because micro- and nanometer-sized particles undergo continuous Brownian motion in solution, the amplitude of the scattered field continuously evolves over time. The detected intensity autocorrelation function can be related to the translational diffusion coefficient *D*, which is related to the hydrodynamic size of the system dispersed in solution. Hence, DLS measures the time dependence of scattering intensity, which provides the hydrodynamic size of the sphere with equivalent diffusion coefficient. The sensitivity of DLS to the larger particles can allow detection of aggregates, and this sensitivity can prevent detection of smaller particles, too. However, if the sample is monodispersed enough and no aggregation phenomenon appears, both the size and the concentration of nanoparticles can be obtained. A similar approach has been applied in the past (Vysotskii et al., 2009; Minelli et al., 2019; Austin et al., 2020) to obtain absolute nanoparticle concentration. In the solutions of diatoms investigated, a precipitate appeared after several hours; it follows that the counts monitored by the DLS system could reveal the rate of precipitation and, hence, a sedimentation rate. To optimize the measurements, several trials were performed with different diatom nominal concentrations, in order to obtain the best reproducibility in the observation of changes of counts during measurements. We optimized and then fixed the attenuator in order to monitor the counts without any verifiable filter. For all measured samples, and for each investigated time, data represented the average of at least five different autocorrelation functions. Cells were sampled during the exponential phase, diluted to obtain approximately 5 × 10^5^ cells in 1 ml of culturing medium, and loaded into a 1 cm path quartz cuvette.

**Figure 1:**
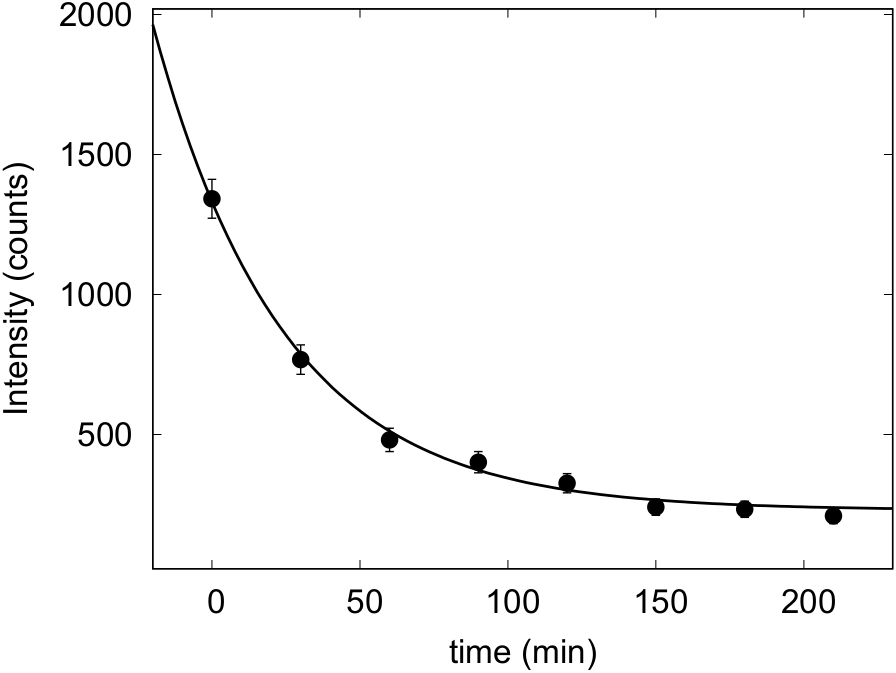
Photon counts as revealed by DLS as a function of time of living cells of *P. tricornutum*—grown at 60 µmol photons·m^−2^ s^−1^ in their growth medium.

The autocorrelation functions, which provided particle size distributions in good agreement with microscopy information, were checked. During the investigated time, form changes of autocorrelation functions were not evident. On the other side, data corresponding to the photon counts on the detector decreased as a function of time, as reported in **Figure 1**. Values of scattering intensity *I*(*t*), expressed as revealed counts as a function of time *t*, were fitted by a simple exponential function describing sedimentation $I\left(t\right)=I_{0}e^{-\frac{t}{\tau }}+b$ , where *I*_0_ is the number of counts at starting time (*t* = 0), *τ* is a constant responsible for diatom sedimentation rate, and *b* is a background. The obtained *τ* values are related to diatom sedimentation rates, obtained for three biological replicas.

### 2.5 Morphological characterization

Diatoms collected during the exponential phase were analyzed with the imaging flow cytometer (IFC) FlowSight^®^ (Amnis Corp., Seattle, WA) using the INSPIRE software package (Amnis Corp.) to assess morphological characteristics. A volume of 10 ml of culture was analyzed within 24 h after sampling. IFC data of more than 50,000 objects present in the samples were saved. Details and settings for the IFC data acquisition were as follows: 10 µm core size diameter, 132 mm/s speed, and 20× magnification; bright field data were collected in channel 1 (LED intensity 35.46 mW), and chloroplast autofluorescence data were collected in channel 5 (642 nm, laser 2 mW). Post-acquisition data analysis was performed using the IDEAS software package following the procedure illustrated in Petrucciani et al. (2022a). The morphological features considered for the analysis of centric species were height, width, area, circularity, diameter, perimeter (quantification of cell circumference), and compactness (degree of how objects are packed together) (IDEAS User Manual, version 6.0, March 2013). For the pennate species, height, width, area, length, elongatedness, perimeter, and compactness were considered. Output numbers refer to the average value of the cited features and are calculated on at least 50,000 cells for each biological replica; they are then used for further statistical analysis.

### 2.6 Frustule characterization

Diatom frustules of *C. muelleri* were obtained through oxidation of the organic material using HCl-KMnO_4_ (modified from Friedrichs et al., 2012). Salts of the culture medium were washed three times with deionized water and then 1.5 ml of a supersaturated solution of KMnO_4_ was added to 1.5 ml of cell suspension. The final solution was incubated overnight at room temperature. A volume of 3 ml of HCl was slowly added to the suspension and then samples were moved to a warming bath at 100°C for 40 min. Finally, the material was washed four times with deionized water to carefully remove the acidic solution. Stubs for SEM observation and analysis were prepared as described in Petrucciani et al. (2022b). Pictures were taken with different orders of magnitude to also obtain morphometrical measurements of frustule details (setae and punctae in *C. muelleri*, Reinke, 1984).

### 2.7 Carbon and nitrogen analysis

Cellular C and N contents were determined in exponentially growing cells using an elemental analyzer (ECS 4010, Costech Italy) connected to the ID Micro EA isotope ratio mass spectrometer (Compact Science Systems, LymedaleBusiness Centre, Newcastle-Under-Lyme, United Kingdom) to obtain C and N stable isotope (δ^13^C and δ^15^N) ratios as reported in Petrucciani et al. (2022b). Data acquisition and analysis were performed with the software EA IsoDelta (Compact Science Systems, LymedaleBusiness Centre, Newcastle-Under-Lyme, United Kingdom). All the measurements were carried out on three biological replicas.

### 2.8 Statistical analysis

One-way analysis of variance (ANOVA), followed by Tukey’s *post-hoc* test, was used to test significant differences among the means of growth rates in three different growing light (independent variable). Two-tailed *t*-test was used to compare dependent variables between two different growing lights (independent variable). Tests were performed with GraphPad prism 8.0.2.263 (GraphPad Software, San Diego, CA, USA) with a level of significance set at 0.05.

Principal component analysis (PCA) was done using PAST 4.03 (Hammer et al., 2001; PAST: Paleontological statistics software package for education and data analysis). Average values of the different morphological features of single cells obtained through IFC analysis were used as dependent variables for PCA; the distinct species and the different light intensities were used as independent variables. Data were normalized using *z*-values ((*n*-mean)/SD). All the results of the statistical analysis are presented in **Supplementary Table 4**.

## 3 Results

### 3.1 Growth analysis

**Figure 2:**
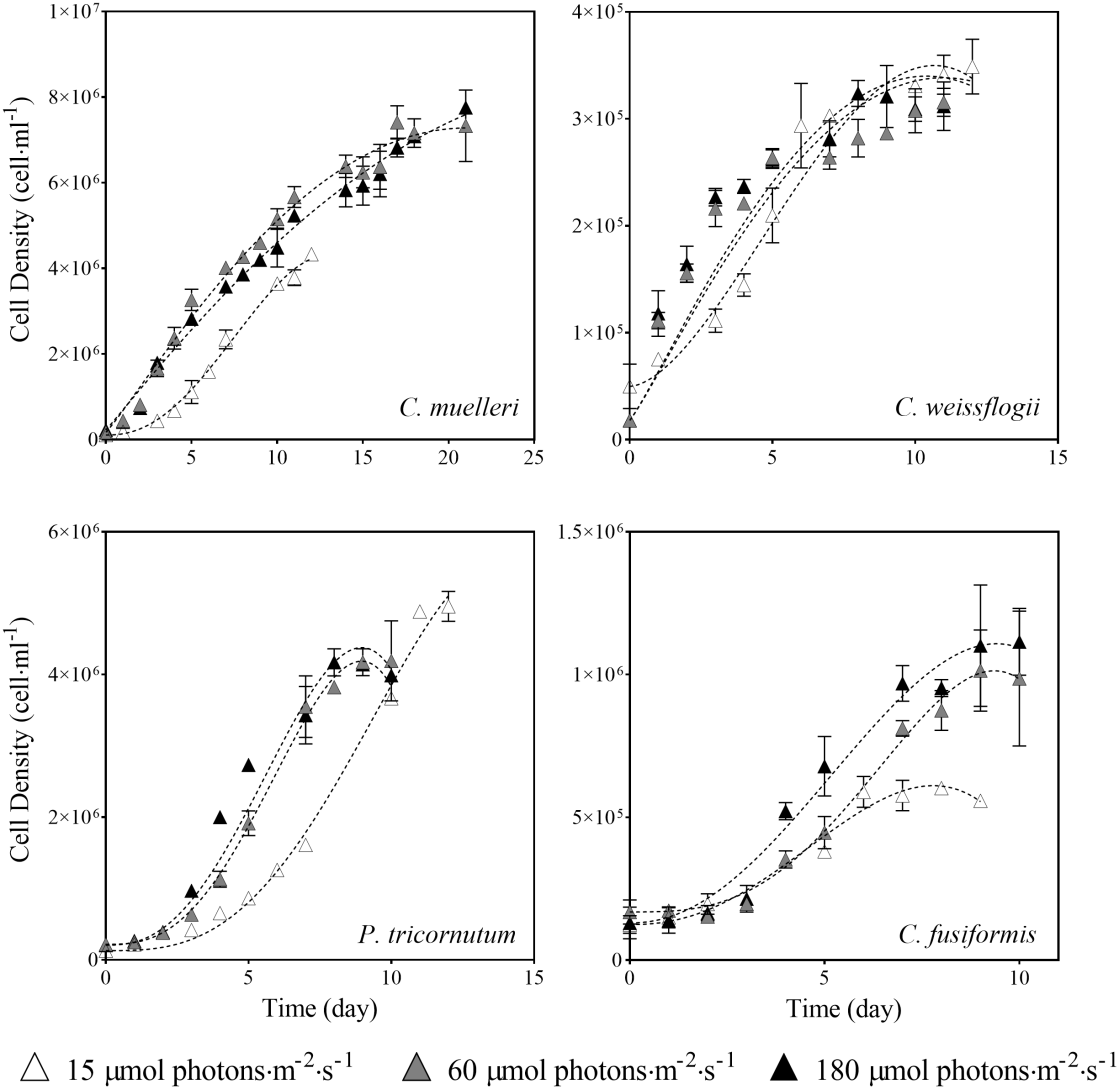
Growth curves of the four diatoms acclimated to different light intensities. Data are means of three biological replicas. Error bars show SD (when not evident, error bars are inside experimental points size). Dashed lines represent the results of the β-function model for each experimental condition (Eq. 1).

Data presented in **Figure 2** and **Table 1** detail the growth of the four diatoms acclimated to increasing light intensities (15, 60, and 180 μmol photons·m^−2^ s^−1^). Growth of *C. muelleri, C. weissflogii*, and *P. tricornutum* was significantly limited by the lowest light intensity (15 μmol photons·m^−2^ s^−1^, **Table 1**). On the other hand, the growth rate of *C. fusiformis* was similar among the three conditions, even though the number of cells reached by *C. fusiformis* grown at 15 μmol photons·m^−2^ s^−1^ was half the ones observed in the other two conditions (**Figure 2**). To address the aim of this work and to avoid the effect of growth limitation on diatom sinking behavior, further investigation focuses on cells acclimated to 60 and 180 μmol photons·m^−2^ s^−1^.

**Table 1:** Average ± SD of specific growth rate (***μ***_max_) in the four diatoms acclimated to different growth lights (***n*** ≥ 3).

		Growth Light (μmol photons·m^−2^·s^−1^)		
		15	60	180
Growth rate (*μ*_max_, day^−1^)	*C. muelleri*	0.22 ± 0.01^a^	0.53 ± 0.03^b^	0.55 ± 0.06^b^
	*C. weissflogii*	0.23 ± 0.03^a^	0.63 ± 0.04^b^	0.63 ± 0.03 ^b^
	*P. tricornutum*	0.240 ± 0.003^a^	0.306 ± 0.005^b^	0.310 ± 0.001^b^
	*C. fusiformis*	0.26 ± 0.03	0.24 ± 0.02	0.23 ± 0.01

Letters indicate significant difference among conditions in the same species (*p* > 0.05).

### 3.2 Silicon quantification per biovolume

**Figure 3:**
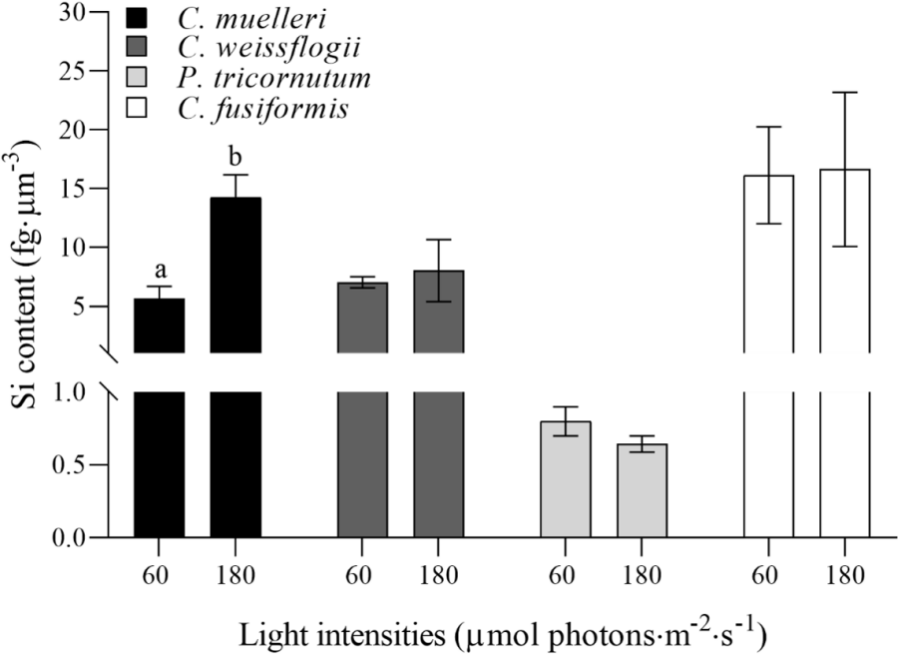
Si content per volume unit (fg·µm^−3^) in the four diatoms acclimated to different growth lights. Data are means of at least three biological replicas. Error bars show SD. Letters represent significant differences between light conditions in the same species (*p* < 0.05).

Si content per biovolume in the four morphologically distinct diatoms, acclimated to different light intensities, is pictured in **Figure 3**. The centric diatom *C. muelleri* was the only species characterized by a notably higher Si content in response to a higher light intensity, while intracellular Si abundance did not significantly change in the other species.Carbon and nitrogen

**Figure 4:**
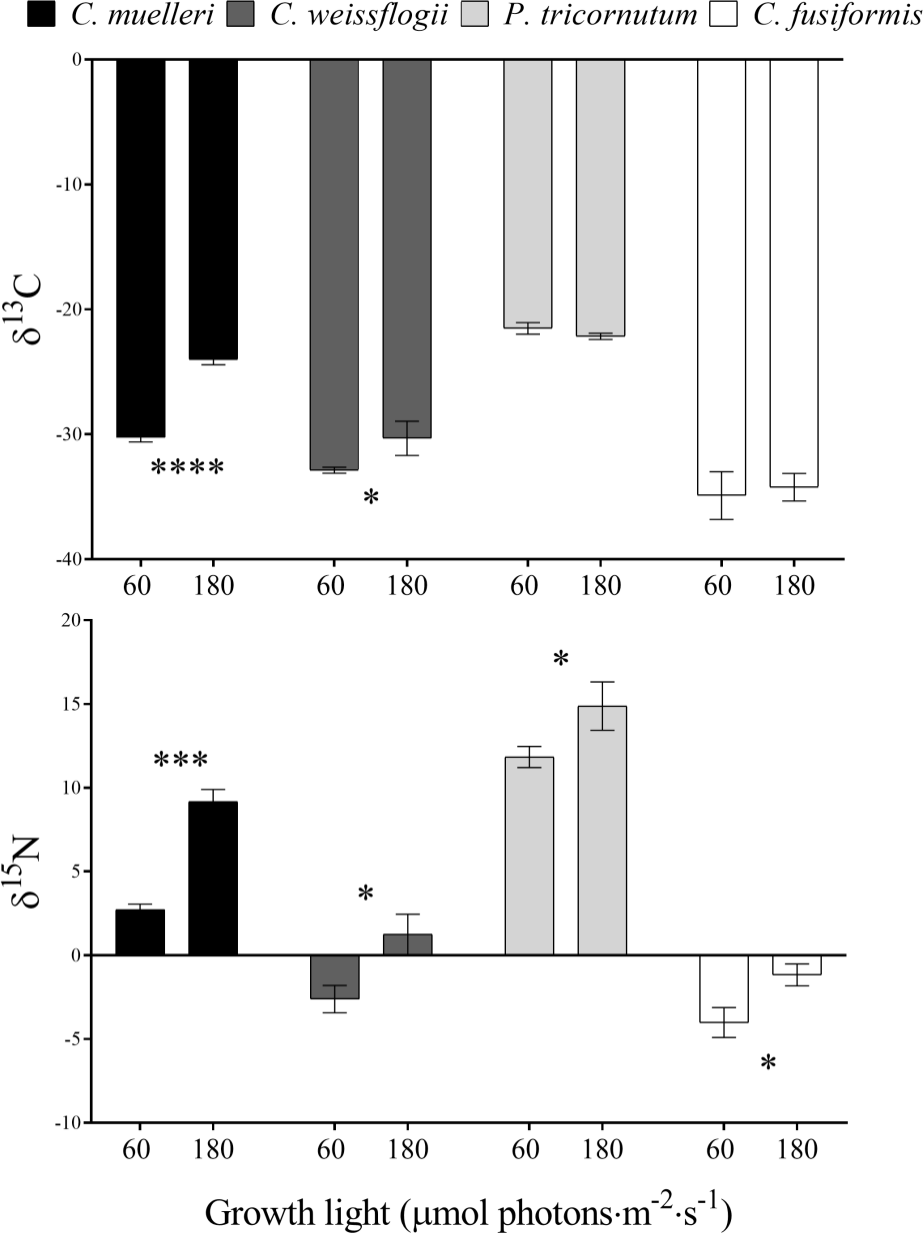
δ^13^C and δ^15^N values in the four diatoms acclimated to different growth lights. Data are means of three biological replicas. Error bars show SD. Asterisks represent significant differences between conditions in the same species (*p < 0.05, ***p < 0.0001, ****p < 0.00001).

The analysis of the elemental quotas per dry weight in the four diatoms acclimated to different light intensities (**Table 2**) revealed no significant change in terms of %C and %N, while *C. weissflogii* showed a lower C/N ratio when grown at lower light intensity. Despite no change in content of the two elements, significant differences were observed in C and N stable isotopic fractionation (**Figure 4**); centric diatoms showed a less negative δ^13^C when acclimated to higher light intensity, especially in *C. muelleri*. Nevertheless, in pennate diatoms, no difference was observed. On the other hand, all the species showed a significant increase in δ^15^N value when acclimated to higher light intensity.

**Table 2:** Elemental C and N quotas and C-to-N ratio of the four diatoms acclimated to different growth lights.

		Growth Light (μmol photons·m^−2^·s^−1^)	
		60	180
% Carbon	C. muelleri	37.6 ± 0.8	36 ± 2
	C. weissflogii	46 ± 1	43 ± 4
	P. tricornutum	54 ± 2	54.9 ± 0.6
	C. fusiformis	52 ± 2	53 ± 2
% Nitrogen	C. muelleri	5.1 ± 0.6	5.8 ± 0.4
	C. weissflogii	6.4 ± 0.5	6.8 ± 0.3
	P. tricornutum	6.9 ± 0.2	6.85 ± 0.09
	C. fusiformis	7.5 ± 0.2	7.2 ± 0.4
C/N	C. muelleri	6.4 ± 0.3	7.3 ± 0.6
	C. weissflogii*	6.3 ± 0.3	7.3 ± 0.4
	P. tricornutum	7.8 ± 0.2	8.0 ± 0.1
	C. fusiformis	6.9 ± 0.2	7.3 ± 0.2

Data are means of three biological replicas ± SD. Asterisks represent significant differences between conditions in the same species (*p* < 0.05).

### 3.3 Morphological characterization

**Figure 5:**
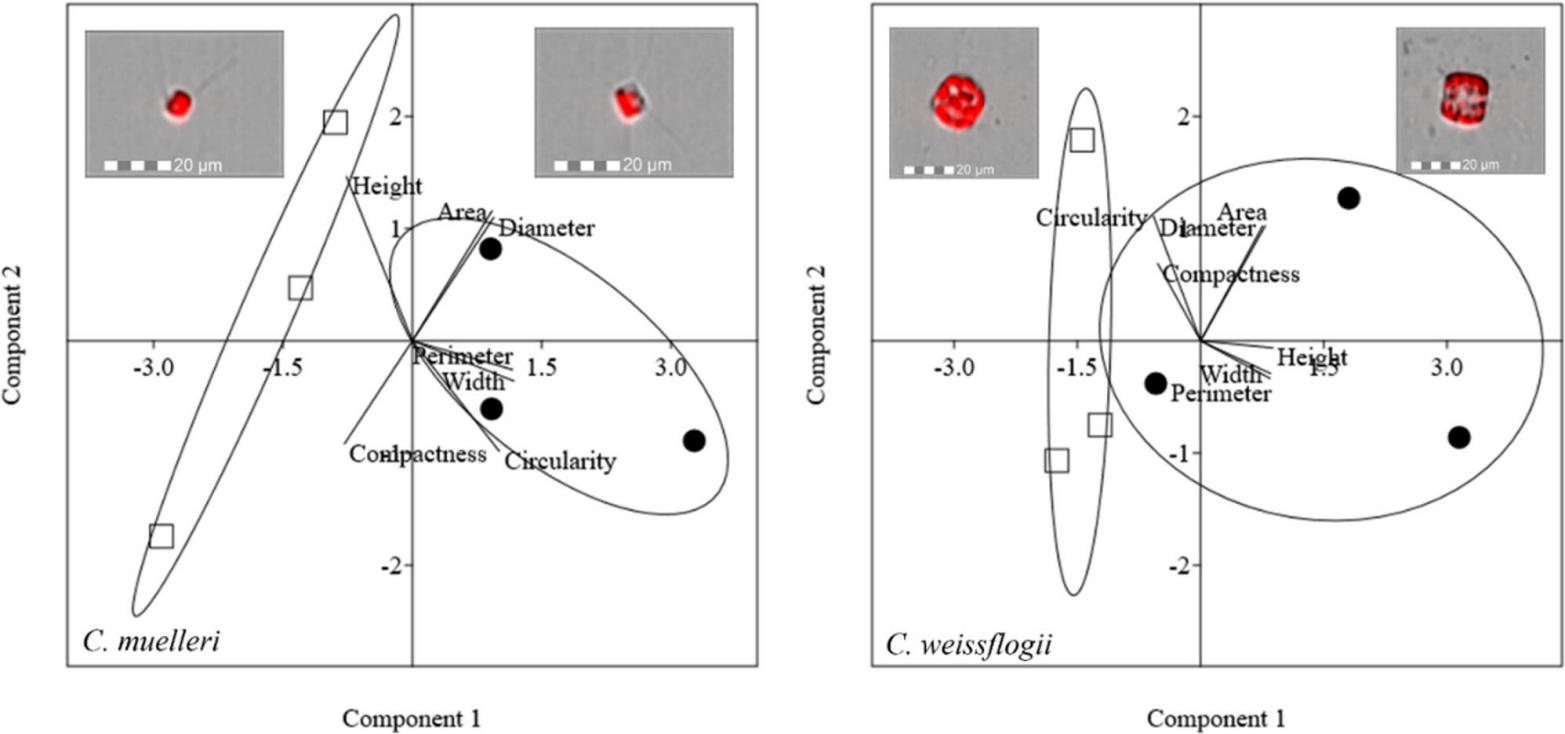
PCA on morphological characteristics of centric diatoms (*C. muelleri* and *C. weissflogii*). Different symbols indicate different growth lights: □ 60 µmol photons·m^−2^·s^−1^ ● 180 µmol photons·m^−2^·s^−1^. Images representing cells acclimated to different light intensities and located close to the respective cluster were obtained by FlowSight**^®^** (Amnis Corp., Seattle, WA), merging the bright field, collected in channel 1, and red chloroplast autofluorescence in channel 5 (details in the Morphological characterization section under Materials and Methods).

Morphological features of centric diatoms (circularity, area, width, height, perimeter, diameter, and compactness) are presented by PCA (**Figure 5**). In *C. muelleri*, PC1 + PC2 explained 91.96% of the total variation contained in the data matrix with PC1 accounting for 66.57% and PC2 accounting for 25.38%. Cells acclimated to different light intensities were differentiated according to PC1, indicating a change in width and perimeter. In *C. weissflogii*, PCA explained 88.99% of the total variation (65.04% + 23.94%, PC1 + PC2) and highlighted a slight change in height according to PC1 in cells acclimated to different growth lights.

**Figure 6:**
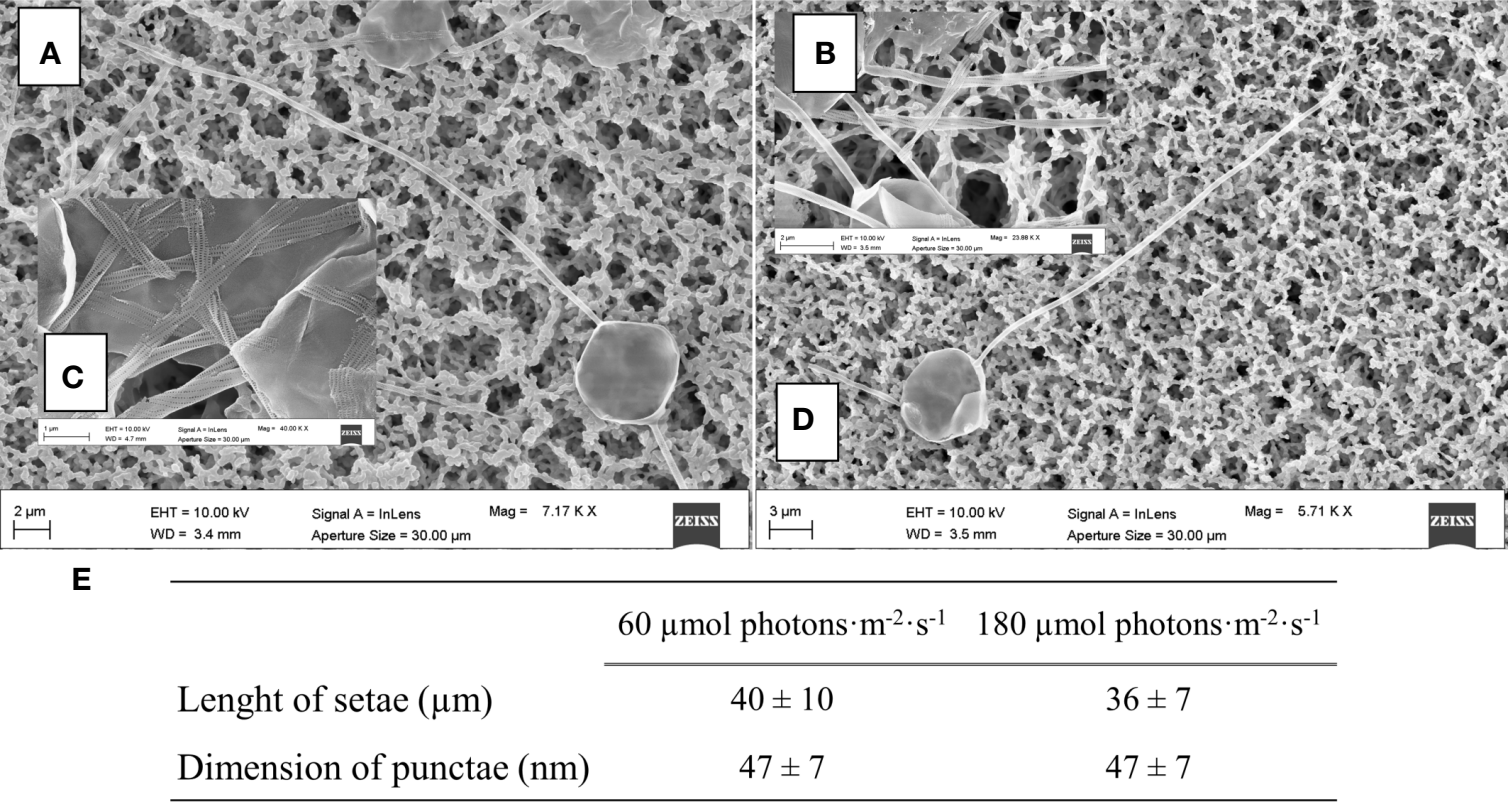
SEM images of centric diatoms (*C. muelleri*) frustules acclimated to 60 μmol photons·m^−2^·s^−1^ **(A)** and 180 µmol photons·m^−2^·s^−1^ **(D)** conditions. Details of *C*. *muelleri* setae at 60 µmol photons·m^−2^·s^−1^ **(B)** and 180 µmol photons·m^−2^·s^−1^ **(C)** are shown and mean values ± SD (*n >*10) are presented in **(E)**.

To complete morphological characterization of the setae bearing diatom, frustules of *C. muelleri* were also characterized by scanning electron microscopy. No significant change was observed in response to light (**Figure 6**).

**Figure 7:**
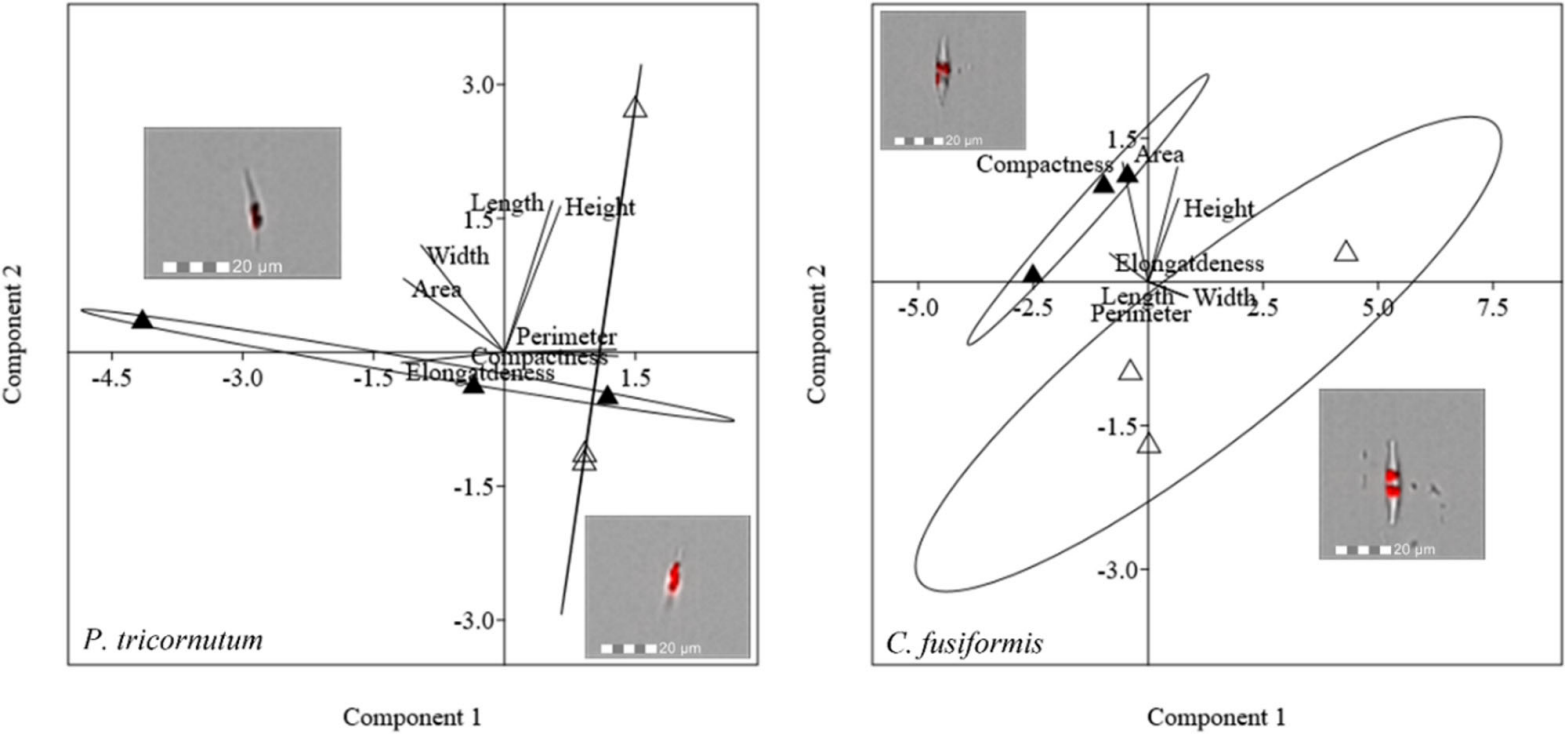
PCA on morphological characteristics of pennate diatoms (*P. tricornutum* and *C. fusiformis*). Different symbols indicate different growth lights:Δ 60 µmol photons·m^−2^·s^−1^ ▲ 180 µmol photons·m^−2^·s^−1^. Images representing cells acclimated to different light intensities and located close to the respective cluster were obtained by FlowSight**^®^** (Amnis Corp., Seattle, WA), merging the bright field, collected in channel 1, and red chloroplast autofluorescence in channel 5 (details in the Morphological characterization section under Materials and Methods).

Morphological characterization of pennate diatoms through PCA (elongatedness, area, width, height, perimeter, length, and compactness) is pictured in **Figure 7**. In *P. tricornutum*, 95.43% of the total variance was explained by PC1 + PC2; the weight of PC1 was 64.72%, while that of PC2 was 30.71%. PCA did not show a net division between cells acclimated to different lights for this species. In *C. fusiformis*, PCA explained 92.17% of the total variance (PC1, 74.65% and PC2, 17.52%). Cells acclimated to different light intensities were separated according to both PC1 and PC2, indicating a change in width and area.

### 3.4 Sinking capacity

**Figure 8:**
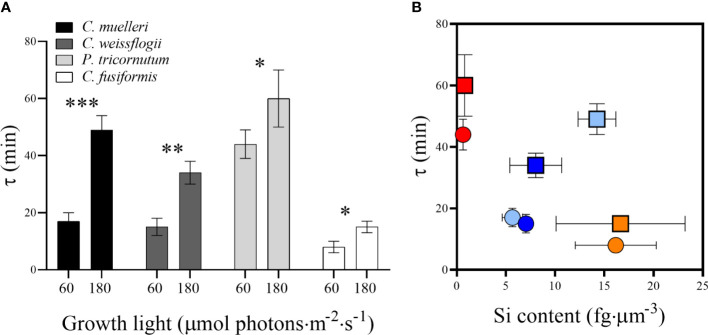
**(A)** *τ* values representing the sedimentation time (min) in diatoms acclimated to different growth lights. Data are means of at least three biological replicas. Error bars show SD. Asterisks represent significant differences between conditions in the same species (*p < 0.05, **p < 0.01, ***p < 0.001). **(B)** *τ* values related to Si content per biovolume in the four diatoms acclimated to 60 µmol photons·m**^−^** ^2^ s**^−^** ^1^ (circles) and 180 µmol photons·m^−2^ s^−1^ (squares). Centric species are shown by light blue (*C. muelleri*) and dark blue (*C. weissflogii*) symbols while pennate species are shown by red (*P. tricornutum*) and orange (*C. fusiformis*) symbols. Error bars show SD (when not evidenced, error bars are inside experimental points size).

Sinking measurements highlighted a direct relation between the light intensity used to grow diatoms and the buoyancy: in particular, in all the species, the fitted value of the parameter *τ* was significantly higher when growth light was higher, as compared to the parameter in low light growing cells; the difference was more evident in the centric species (**Figure 8A**). Because the *τ* parameter is related to the necessary time to sink, clearly related to chemical physical properties of each particular diatom, it is inversely proportional to sedimentation rate. We considered the recent results obtained by Hamano et al. (2021), where four different diatom species’ experimental velocity rates had been estimated by a sophisticated homemade optical microscope system. In particular, they investigated the sinking rate of *P. tricornutum* irradiated with a white fluorescent lamp of 85 µmol photons·m**^−^** ^2^ s**^−^** ^1^ for 12 h every day, which we assimilated to our *P. tricornutum* grown with the irradiation of 60 µmol photons·m**^−^** ^2^ s**^−^** ^1^. Because the sedimentation rate has to be inversely proportional to the *τ* parameter, a numerical coefficient was calculated correlating *P. tricornutum* vertical velocity obtained by Hamano et al. (2021) to the estimated *τ* for the same species at the lower irradiation (details are reported in the caption of **Table S3**). Consequently, sedimentation rates are reported in **Table S3**. By plotting τ values and the relative Si contents per biovolume (**Figure 8B**), at low light, the higher was the silicification, the lower was the τ parameter, contrary to what observed for other measured parameters (**Figure S3**). When the energy given to cells was higher, the trend was partially lost.

## 4 Discussion

Buoyancy and buoyancy control are crucial to vertical movements: they allow a rapid escape from predators and provide a way to retrieve essential resources such as light and nutrients (Raven and Waite, 2004; Gemmell et al., 2016; Lavoie and Raven 2020; Du Clos et al., 2021; Petrucciani et al., 2022a). Shape, size, and silicification have been deeply investigated as factors affecting diatom ability to move in the water column. The closer the cell geometry is to the elongated shape, the slower the sinking rate is observed (Durante et al., 2019). Shape being equal, silicification and size drive cell density and hence the diatom sinking rate (Raven and Waite, 2004). Furthermore, it has been observed that diatoms’ sinking rate depends on light availability (Bienfang, 1981; Bienfang et al., 1983), which can then be ascribed among the factors regulating cell density (Gemmell et al., 2016).

### 4.1 Morphology and silicification affecting buoyancy

According to results, silicification was the major driver affecting buoyancy as compared to size, shape and weight: indeed, frustule density expressed as Si content per volume was inversely related to the sedimentation time (**Figure 8B**). The same relation was not observed in the case of Si content per cell, dry weight, and cellular volume (**Figure S3**). Indeed, *C. fusiformis* was the most silicified species per unit volume (**Figure 3**) among the experimental ones, and it was the fastest to sink down even though not the biggest or the heaviest. Moreover, this species is known to secrete extracellular polymeric substances (EPS) to assist in surface attachment, providing adhesion and aggregation among cells (Tong and Derek, 2021) and contributing to rapid sinking rate (Ploug and Grossart, 2000; Lavoie et al., 2016; Laurenceau-Cornec et al., 2020).

Our experimental data confirmed that elongated shape favors cell floating as proposed by Durante et al. (2019): the sedimentation time of the pennate *P. tricornutum* was significantly higher than the time of the centric *C. muelleri*, despite a similar cell volume (**Figures 8A**, **S3** and **Table S3**). On the other hand, distantly sized diatoms (*C. muelleri* and *C. weissflogii*) with the same geometry showed a similar sedimentation time, confirming that there is no obligate relation between cell morphology and sinking rate for metabolically active cells (**Figure S3**) (Waite et al., 1997; Gemmell et al., 2016). The response pattern observed changed according to light intensity (**Figure 8B**), suggesting that the higher energy availability to high-light-grown cells altered the major role of silicification in controlling buoyancy.

Direct observation of sinking capacity in diatoms was carried out for the first time through DLS analysis, allowing a continuous record of cells’ sedimentation from the top section without perturbing the water column. We underline that the results presented here are the average of at least three replicas and further measurements were performed at higher diatom concentrations, providing the same *τ* parameters. Given the importance to directly assess sinking capacity in phytoplankton, numerous efforts were made to figure out a suitable method (Walsby and Holland, 2006 and reference therein; Gemmell et al., 2016; Bannon and Campbell, 2017; Hamano et al., 2021); the present study confirms the feasibility of using DLS to achieve this purpose.

### 4.2 Light-dependent buoyancy control

In this study, the two light conditions applied in the sinking experiments (60 and 180 μmol photons·m**^−^** ^2^ s**^−^** ^1^) were not limiting growth (**Figure 2** and **Table 1**): in fact, growth was limited at 15 μmol photons m**^−^** ^2^ s**^−^** ^1^. In addition, the highest experimental light was not photo-damaging since the growth rate and photosynthetic efficiency of cells acclimated to 180 μmol·photons m**^−^** ^2^ s**^−^** ^1^ were similar to those of cells acclimated to 60 μmol·photons m**^−^** ^2^ s**^−^** ^1^, respectively (Conn et al., 2004; **Figures S1, S2**). Therefore, the greater time required to sink (i.e., enhanced floating capacity), which was observed in high-light-acclimated cells, was not a way to move towards optimal irradiance (and besides, not even a way to escape from excess irradiance) (**Figure 8A** and **Table S3**). Moreover, buoyancy was independent of the Si content, suggesting the existence of a control at the cellular level, which was regulated by light and not by silicification (**Figures 3**, **8**).

A higher light availability means a higher energy availability to cells, which can be converted into metabolic energy *via* photosynthesis and activates energy-dependent mechanisms, also enhancing diatom floating (Waite et al., 1997; Lavoie and Raven, 2020; Du Clos et al., 2021) as we observed (**Figure 8**).

Cell growth, C and N quotas, and cellular volume of all the species were not affected by the light variation (**Table 2**). Overall, we did not record a drastic morphological change that could explain a change in sedimentation rate (**Figures 5**, **7** and **Table S1**). Also, frustules did not show significant changes in ultrastructure (**Figure 6**). None of these parameters thus suggest/explain which mechanisms could be involved in buoyancy control except for the observed isotopic fractionation. In fact, δ^13^C values were affected by light in centric diatoms, which showed a higher difference in *τ* values in response to light intensity (**Figure 4**). A change in C fractionation could be due to a shift in inorganic C source (Vuorio et al., 2006); in particular, higher light intensity led to decreased fractionation and therefore an increased use of HCO_3_^−^ occurred during photosynthesis. A CO_2_ concentrating mechanism was therefore activated when more energy was available (Riebesell et al., 2000; Giordano et al., 2005; Petrucciani et al., 2022b). Moreover, all diatom species decreased N fractionation in response to higher light intensity (**Figure 4**), suggesting that the higher supply of energy provided more reducing power, which, in turn, was exploited in the assimilation of more costly N species (Needoba et al., 2003; Needoba and Harrison, 2004). Since the presence of the cited processes is consistent with a change in photosynthesis-to-photorespiration ratio (Giordano et al., 2005), we cannot exclude the fact that gas release may play a role in controlling sinking rate/floating capacity.

According to literature, other energy-dependent mechanisms active in diatoms that control buoyancy are possibly the modulation of vacuolar solution density and a variable fraction of the cell volume occupied by the vacuole (Lavoie and Raven, 2020 and references therein). The density of the frustule in diatoms is indeed countered by the presence of vacuoles, whose density is supposed to be rapidly modulated in three ways: (i) high-frequency modulation of Na^+^ and K^+^ permeability by selective ion transport; (ii) metabolic interconversion of low-density cations and high-density organic cations; and (iii) fast cyclical changes in the cell expansion rate (i.e., active water transport and cytoskeletal motors) (Raven and Doblin, 2014; Lavoie and Raven, 2020). The last one is the most energetically convenient according to Lavoie and Raven (2020). Our data that showed a small morphological change in cell height and area (**Figures 5**, **7**) and did not record a change in K^+^ cell content between cells acclimated to low and high light intensity (**Table S2**) support the third strategy.

In conclusion, the direct observation of diatom sinking behavior has elucidated how each of the addressed factors, commonly investigated individualistically, is involved in buoyancy and in its control relatively to the others. When considering diatom biodiversity, silicification controls the sinking rate more than morphology and a light-driven response. When energy is more available though, buoyancy is tuned by cell metabolism as strongly observed in *C. muelleri* showing a lower sinking rate despite enhanced frustule deposition.

## Data availability statement

The original contributions presented in the study are included in the article/**Supplementary Material**. Further inquiries can be directed to the corresponding author.

## Author contributions

AP carried out all the experiments and analyzed the data. PM performed DLS measurements. PM and MGO analyzed and discussed DLS data. AN conceived and designed the project. AP and AN wrote the paper. PM and MGO contributed to the final version. All authors contributed to the article and approved the submitted version.

## Footnotes

1
https://www.osram.com/ecat/LUMILUX%20T8-Fluorescent%20lamps%20T8-Fluorescent%20lamps-Lamps-Digital%20Systems/com/en/GPS01_1027891/ZMP_60436/.

## References

[B1] Allen W. E. (1932). Problems of flotation and deposition of marine plankton diatoms. Trans. Am. Micros. Soc 51, 1–7. doi: 10.2307/3222044

[B2] Andreozzi P. Ricci C. Porcel J. E. M. Moretti P. Di Silvio D. Amenitsch H.. (2019). Mechanistic study of the nucleation and conformational changes of polyamines in presence of phosphate ions. J. Colloid Interface Sci. 543 2019, 335–342. doi: 10.1016/j.jcis.2019.02.040 30831359

[B3] Armbrust E. V. (2009). The life of diatoms in the world’s oceans. Nature 459, 185–192. doi: 10.1038/nature08057 19444204

[B4] Arrieta J. Jeanneret R. Roig P. Tuval I. (2020). On the fate of sinking diatoms: the transport of active buoyancy-regulating cells in the ocean. Phil. Trans. R. Soc A. 378(2179). doi: 10.1098/rsta.2019.0529 PMC7422867 32762433

[B5] Austin J. Minelli C. Hamilton D. Wywijas M. Jones H. J. (2020). Nanoparticle number concentration measurements by multi-angle dynamic light scattering. J. Nanoparticle Res. 22, 108. doi: 10.1007/s11051-020-04840-8

[B6] Bannon C. C. Campbell D. A. (2017). Sinking towards destiny: High throughput measurement of phytoplankton sinking rates through time-resolved fluorescence plate spectroscopy. PloS One 12, e0185166. doi: 10.1371/journal.pone.0185166 28972987 PMC5626032

[B7] Berne B. J. Pecora R. (1976). Dynamic light scattering with applications to chemistry, biology, and physics (New York: John Wiley and Sons, a Wiley Interscience Publication).

[B8] Bienfang P. K. (1981). Sinking rate dynamics of *Crzcosphaera carterae* braarud. i. effects of growth rate, limiting substrate, and diurnal variation in steady-state populations. J. Exp. Mar. Biol. Ecol. 49, 217–233. doi: 10.1016/0022-0981(81)90072-1

[B9] Bienfang P. Szyper J. Laws E. (1983). Sinking rate and pigment responses to light-limitation of a marine diatom - implications to dynamics of chlorophyll maximum layers. Oceanologica Acta 6 (1), 55–62.

[B10] Bowler C. De Martino A. Falciatore A. (2010). Diatom cell division in an environmental context. Curr. Opin. Plant Biol. 13, 623–630. doi: 10.1016/j.pbi.2010.09.014 20970371

[B11] Brzezinski M. Olson R. Chisholm S. (1990). Silicon availability and cell-cycle progression in marine diatoms. Mar. Ecol. Prog. Ser. 67, 83–96. doi: 10.3354/meps067083

[B12] Conn S. A. Bahena M. Davis J. T. Ragland R. L. Rauschenberg C. D. Smith B. J. (2004). Characterisation of the diatom photophobic response to high irradiance. Diatom Res. 19, 167–179. doi: 10.1080/0269249X.2004.9705869

[B13] Du Clos K. T. Karp-Boss L. Gemmell B. J. (2021). Diatoms rapidly alter sinking behavior in response to changing nutrient concentrations. Limnol Oceanogr 66, 892–900. doi: 10.1002/lno.11649

[B14] Du Clos K. T. Karp-Boss L. Villareal T. A. Gemmell B. J. (2019). *Coscinodiscus wailesii* mutes unsteady sinking in dark conditions. Biol. Lett. 15, 20180816. doi: 10.1098/rsbl.2018.0816 30890072 PMC6451371

[B15] Durante G. Basset A. Stanca E. Roselli L. (2019). Allometric scaling and morphological variation in sinking rate of phytoplankton. J. Phycol. 55, 1386–1393. doi: 10.1111/jpy.12916 31483867

[B16] Durbin E. G. (1977). Studies on the autecology of the marine diatom *Thalassiosira nordenskioeldii.* II. the influence of cell size on growth rate, and carbon, nitrogen, chlorophyll *a* and silica content. J. Phycol. 13 (2), 150–155. doi: 10.1111/j.1529-8817.1977.tb02904.x

[B17] Falciatore A. d'Alcalà M. R. Croot P. Bowler C. (2000). Perception of environmental signals by a marine diatom. Science 288 (5475), 2363–2366. doi: 10.1126/science.288.5475.2363 10875921

[B18] Fanesi A. Raven J. A. Giordano M. (2014). Growth rate affects the responses in the green alga tretaselmis suecica to the external perturbation. Plant Cell Environ. 37 (2), 512–519. doi: 10.1111/pce.12176 23927015

[B19] Flynn K. J. Martin-Jezequel V. (2000). Modelling Si-n-limited growth of diatoms. J. Plankton Res. 22, 447–472. doi: 10.1093/plankt/22.3.447

[B20] Friedrichs L. Maier M. Hamm C. (2012). A new method for exact three-dimensional reconstruction of diatom frustules. J. Microscopy 248 (2), 208–217. doi: 10.1111/j.1365-2818.2012.03664.x 23078119

[B21] Gemmell B. J. Oh G. Buskey E. J. Villareal T. A. (2016). Dynamic sinking behaviour in marine phytoplankton: rapid changes in buoyancy may aid in nutrient uptake. Proc. R. Soc B. 283, 20161126. doi: 10.1098/rspb.2016.1126 PMC5069504 27708154

[B22] Giordano M. Beardall J. Raven J. A. (2005). CO_2_ concentrating mechanisms in algae: mechanisms, environmental modulation, and evolution. Annu. Rev. Plant Biol. 56, 99–131. doi: 10.1146/annurev.arplant.56.032604.144052 15862091

[B23] Hamano R. Shoumura S. Takeda Y. Yamazaki T. Hirayama K. Hanada Y.. (2021). Sinking of four species of living diatom cells directly observed by a “tumbled” optical microscope. Microsc Microanal 27, 1154–1160. doi: 10.1017/S1431927621012150 34294188

[B24] Hammer O. Harper D. A. T. Ryan P. D. (2001). PAST: paleontological statistics software package for education and data analysis. Palaeontol. Electron. 4:9

[B25] Hervé V. Derr J. Douady S. Quinet M. Moisan L. Lopez P. J. (2012). Multiparametric analyses reveal the ph-dependence of silicon biomineralization in diatoms. PloS One 7, e46722. doi: 10.1371/journal.pone.0046722 23144697 PMC3483172

[B26] Hildebrand M. Lerch S. J. L. Shrestha R. P. (2018). Understanding diatom cell wall silicification-moving forward. Front. Mar. Sci. 5, 125. doi: 10.1093/bbb/zbab069

[B27] Jin X. Gruber N. Dunne J. P. Sarmiento J. L. Armstrong R. A. (2006). Diagnosing the contribution of phytoplankton functional groups to the production and export of particulate organic carbon, CaCO_3_, and opal from global nutrient and alkalinity distributions: diagnosing phytoplankton functional groups. Global Biogeochem. Cycles 20(2). doi: 10.1029/2005GB002532

[B28] Laurenceau-Cornec E. C. Le Moigne F. A. C. Gallinari M. Moriceau B. Toullec J. Iversen M. H.. (2020). New guidelines for the application of stokes' models to the sinking velocity of marine aggregates. Limnol Oceanogr 65, 1264–1285. doi: 10.1002/lno.11388

[B29] Lavoie M. Raven J. A. (2020). How can large-celled diatoms rapidly modulate sinking rates episodically? J. Exp. Bot. 71, 3386–3389. doi: 10.1093/jxb/eraa129 32161972 PMC7364400

[B30] Lavoie M. Raven J. A. Levasseur M. (2016). Energy cost and putative benefits of cellular mechanisms modulating buoyancy in a flagellate marine phytoplankton. J. Phycol. 52, 239–251. doi: 10.1111/jpy.12390 27037589

[B31] Leblanc K. Quéguiner B. Diaz F. Cornet V. Michel-Rodriguez M. Durrieu de Madron X.. (2018). Nanoplanktonic diatoms are globally overlooked but play a role in spring blooms and carbon export. Nat. Commun. 9, 953. doi: 10.1038/s41467-018-03376-9 29507291 PMC5838239

[B32] Malviya S. Scalco E. Audic S. Vincent F. Veluchamy A. Poulain J.. (2016). Insights into global diatom distribution and diversity in the world’s ocean. Proc. Natl. Acad. Sci. U.S.A. 113, E1516–E1525. doi: 10.1073/pnas.1509523113 26929361 PMC4801293

[B33] Margaref R. (1978). Primary partitioning and storage of photosynthate in sucrose and starch in leaves of C4 plants. Oceanologica Acta 1 (4), 493–509. doi: 10.1007/BF00202661

[B34] Marron A. O. Ratcliffe S. Wheeler G. L. Goldstein R. E. King N. Not F.. (2016). The evolution of silicon transport in eukaryotes. Mol. Biol. Evol. 33, 3226–3248. doi: 10.1093/molbev/msw209 27729397 PMC5100055

[B35] Martin-Jézequel V. Hildebrand M. Brzezinski M. A. (2000). Silicon metabolism in diatoms: implications for growth. J. Phycol 36, 821–840. doi: 10.1046/j.1529-8817.2000.00019.x

[B36] Mcnown J. S. Malaika J. (1950). Effects of particle shape in settling velocity at low reynolds numbers. Trans. Am. Geophys. Union 31, 74–82. doi: 10.1029/TR031i001p00074

[B37] Minelli A. Bartczak D. Peters R. Rissler J. Undas A. Sikora A.. (2019). Sticky measurement problem: Number concentration of agglomerated nanoparticles. Langmuir 35 (14), 4927–4935. doi: 10.1021/acs.langmuir.8b04209 30869903

[B38] Moriceau B. Garvey M. Ragueneau O. Passow U. (2007). Evidence for reduced biogenic silica dissolution rates in diatom aggregates. Mar. Ecol. Prog. Ser. 333, 129–142. doi: 10.3354/meps333129

[B39] Needoba J. A. Harrison P. J. (2004). INFLUENCE OF LOW LIGHT AND a LIGHT: DARK CYCLE ON NO3– UPTAKE, INTRACELLULAR NO3–, AND NITROGEN ISOTOPE FRACTIONATION BY MARINE PHYTOPLANKTON. J. Phycology 40, 505–516. doi: 10.1111/j.1529-8817.2004.03171.x

[B40] Needoba J. A. Waser N. A. Harrison P. J. Calvert S. E. (2003). Nitrogen isotope fractionation in 12 species of marine phytoplankton during growth on nitrate. Mar. Ecol. Prog. Ser. 255, 81–91. doi: 10.3354/meps255081

[B41] Padisak J. Soroczki-Pinter E. Rezner Z. (2003). Sinking properties of some phytoplankton shapes and the relation of form resistance to morphological diversity of plankton – an experimental study. Hydrobiologia 500, 243–257. doi: 10.1007/978-94-007-1084-9_18

[B42] Petrucciani A. Chaerle P. Norici A. (2022a). Diatoms versus copepods: could frustule traits have a role in avoiding predation? Front. Mar. Sci. 8. doi: 10.3389/fmars.2021.804960

[B43] Petrucciani A. Knoll A. H. Norici A. (2022b). Si Decline and diatom evolution: Insights from physiological experiments. Front. Mar. Sci. 9. doi: 10.3389/fmars.2022.924452

[B44] Ploug H. Grossart H.-P. (2000). Bacterial growth and grazing on diatom aggregates: Respiratory carbon turnover as a function of aggregate size and sinking velocity. Limnol. Oceanogr 45, 1467–1475. doi: 10.4319/lo.2000.45.7.1467

[B45] Pondaven P. Gallinari M. Chollet S. Bucciarelli E. Sarthou G. Schultes S.. (2007). Grazing-induced changes in cell wall silicification in a marine diatom. Protist 158, 21–28. doi: 10.1016/j.protis.2006.09.002 17081802

[B46] Raven J. A. Doblin M. A. (2014). Active water transport in unicellular algae: where, why, and how. J. Exp. Bot. 65, 6279–6292. doi: 10.1093/jxb/eru360 25205578

[B47] Raven J. A. Waite A. M. (2004). The evolution of silicification in diatoms: inescapable sinking and sinking as escape? New Phytol. 162, 45–61. doi: 10.1111/j.1469-8137.2004.01022.x

[B48] Reinke D. C. (1984). Ultrastructure of *Chaetoceros muelleri* (bacillariophyceae): auxospore, resting spore and vegetative cell morphology. J.Phycology 20, 153–155. doi: 10.1111/j.0022-3646.1984.00153.x

[B49] Reynolds C. S. (2006). Ecology of phytoplankton (Cambridge, UK: Cambridge University Press), 550.

[B50] Riebesell U. Burkhardt S. Dauelsberg A. Kroon B. (2000). Carbon isotope fractionation by a marine diatom: dependence on the growth-rate-limiting resource. Mar. Ecol. Prog. Ser. 193, 295–303. doi: 10.3354/meps193295

[B51] Shrestha R. P. Tesson B. Norden-Krichmar T. Federowicz S. Hildebrand M. Allen A. E. (2012). Whole transcriptome analysis of the silicon response of the diatom *Thalassiosira pseudonana* . BMC Genomics 13, 499. doi: 10.1186/1471-2164-13-499 22994549 PMC3478156

[B52] Smayda T. Y. (1970). The suspension and sinking of phytoplankton in the sea. Oceanogr. Mar. Biol. Ann. Rev. 8, 353–414.

[B53] Smayda T. J. Bienfang P. K. (1983). Suspension properties of various phyletic groups of phytoplankton and tintinnids in an oligotrophic subtropical system. Mar. Ecol. 4, 289–300. doi: 10.1111/j.1439-0485.1983.tb00115.x

[B54] Sommer U. Charalampous E. Genitsaris S. Moustaka-Gouni M. (2016). Benefits, costs and taxonomic distribution of marine phytoplankton body size. J. Plankton Res. 39, 494–508. doi: 10.1093/plankt/fbw071

[B55] Su Y. Lundolm M. Legaard M. (2018). The effect of different light regimes on diatom frustule silicon concentration. Algal Res. 29, 36–40. doi: 10.1016/j.algal.2017.11.014

[B56] Sutton J. N. André L. Cardinal D. Conley D. J. de Souza G. F. Dean J.. (2018). A review of the stable isotope bio-geochemistry of the global silicon cycle and its associated trace elements. Front. Earth Sci. 5. doi: 10.3389/feart.2017.00112

[B57] Tong C. Y. Derek C. J. C. (2021). The role of substrates towards marine diatom *Cylindrotheca fusiformis* adhesion and biofilm development. J. Appl. Phycol 33, 2845–2862. doi: 10.1007/s10811-021-02504-1

[B58] Tréguer P. Bowler C. Moriceau B. Dutkiewicz S. Gehlen M. Aumont O.. (2018). Influence of diatom diversity on the ocean biological carbon pump. Nat. Geosci 11, 27–37. doi: 10.1038/s41561-017-0028-x

[B59] Vrieling E. G. Sun Q. Tian M. Kooyman P. J. Gieskes W. W. C. van Santen R. A.. (2007). Salinity-dependent diatom biosilicification implies an important role of external ionic strength. Proc. Natl. Acad. Sci. 104, 10441–10446. doi: 10.1073/pnas.0608980104 17563373 PMC1965532

[B60] Vuorio K. Meili M. Sarvala J. (2006). Taxon-specific variation in the stable isotopic signatures (δ^13^C and δ^15^N) of lake phytoplankton. Freshw. Biol. 51, 807–822. doi: 10.1111/j.1365-2427.2006.01529.x

[B61] Vysotskii V. V. Uryupina O. Y. Gusel’nikova A. V. Roldugin V. I. (2009). ) on the feasibility of determining nanoparticle concentration by the dynamic light scattering method. Colloid J. 71 (6), 739–744. doi: 10.1134/S1061933X09060027

[B62] Waite A. Fisher A. Thompson P. Harrison P. (1997). Sinking rate versus cell volume relationships illuminate sinking rate control mechanisms in marine diatoms. Mar. Ecol. Prog. Ser. 157, 97–108. doi: 10.3354/meps157097

[B63] Waite A. M. Thompson P. A. Harrison P. J. (1992). Does energy control the sinking rates of marine diatoms? Limnol. Oceanogr. 37, 468–477. doi: 10.4319/lo.1992.37.3.0468

[B64] Walsby A. E. Holland D. P. (2006). Sinking velocities of phytoplankton measured on a stable density gradient by laser scanning. J. R. Soc Interface. 3, 429–439. doi: 10.1098/rsif.2005.0106 16849271 PMC1578759

[B65] Xu H. Shi Z. Zhang X. Pang M. Pan K. Liu H. (2021). Diatom frustules with different silica contents affect copepod grazing due to differences in the nanoscale mechanical properties. Limnol Oceanogr 66, 3408–3420. doi: 10.1002/lno.11887

[B66] Yin X. Goudriaan J. Lantinga E. A. Vos J. Spiertz H. J. (2003). A flexible sigmoid function of determinate growth. Ann. Bot. 91, 361–371. doi: 10.1093/aob/mcg029 12547689 PMC4244967

