# Supplementary material for: Integrative effects of morphology, silicification, and light on diatom vertical movements
Source: Front Plant Sci. 2023 Mar 27;14:1143998. doi: 10.3389/fpls.2023.1143998 (PMC10087530; doi:10.3389/fpls.2023.1143998)
Supplement: Supplementary file 1 [file DataSheet_1.docx]

Supplementary Material

**Integrative effects of morphology, silicification and light on diatom vertical movements**

Petrucciani Alessandra^1^, Moretti Paolo^1^, Ortore Maria Grazia^1^, Norici Alessandra^1^*

^1^ Dipartimento di Scienze della Vita e dell’Ambiente, Università Politecnica delle Marche, Ancona, Italy

*** Correspondence:**Alessandra Norici
a.norici@univpm.it

# Supplementary Figures and Tables


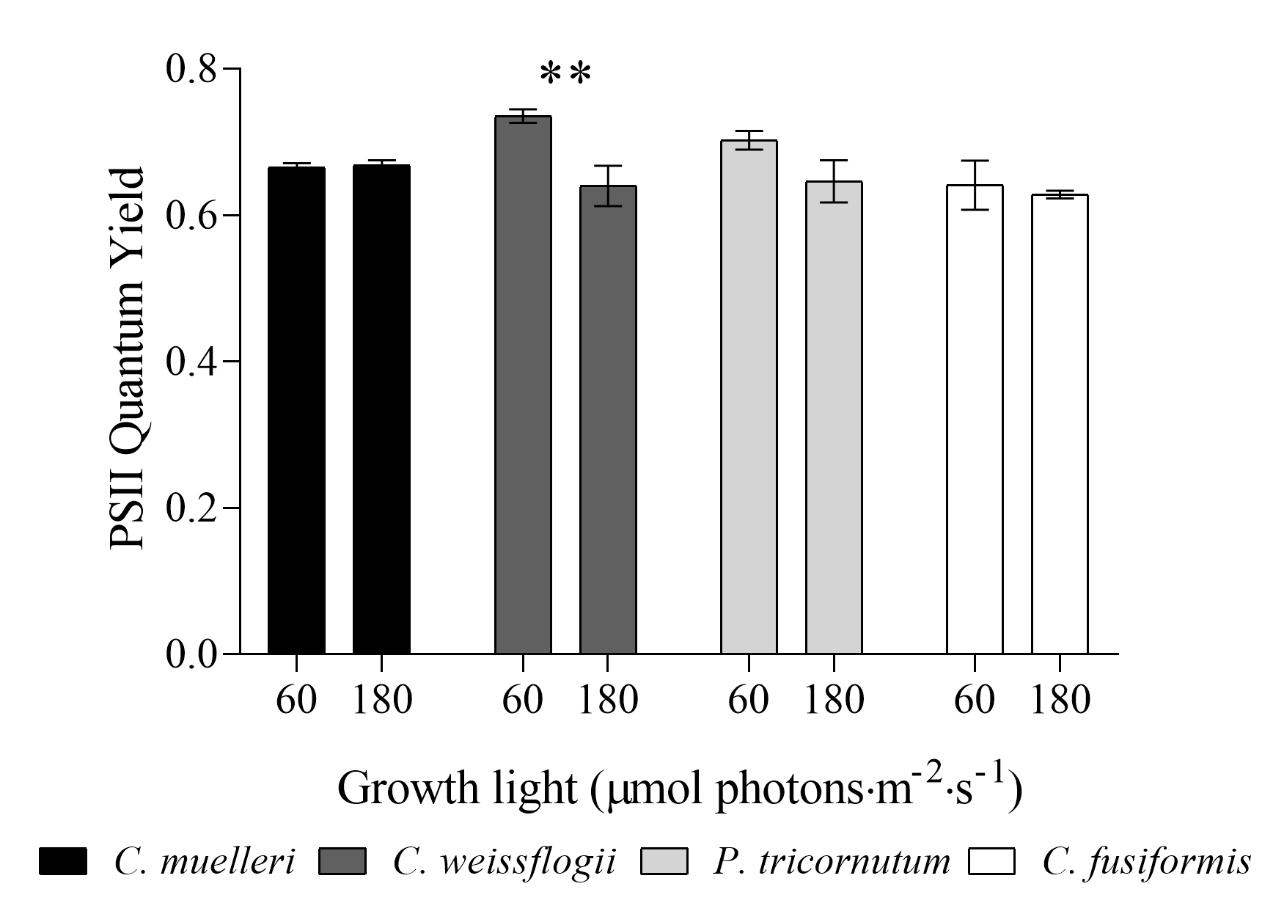


Supplementary Figure 1. PSII Quantum Yield of the four diatoms acclimated to different light intensities. Data are means of 3 biological replicas. Error bars show SD. Asterisks represent significant differences among conditions in the same species (* *p*<0.05, ** *p*< 0.001). Data were obtained as described in Petrucciani et al., 2022b.


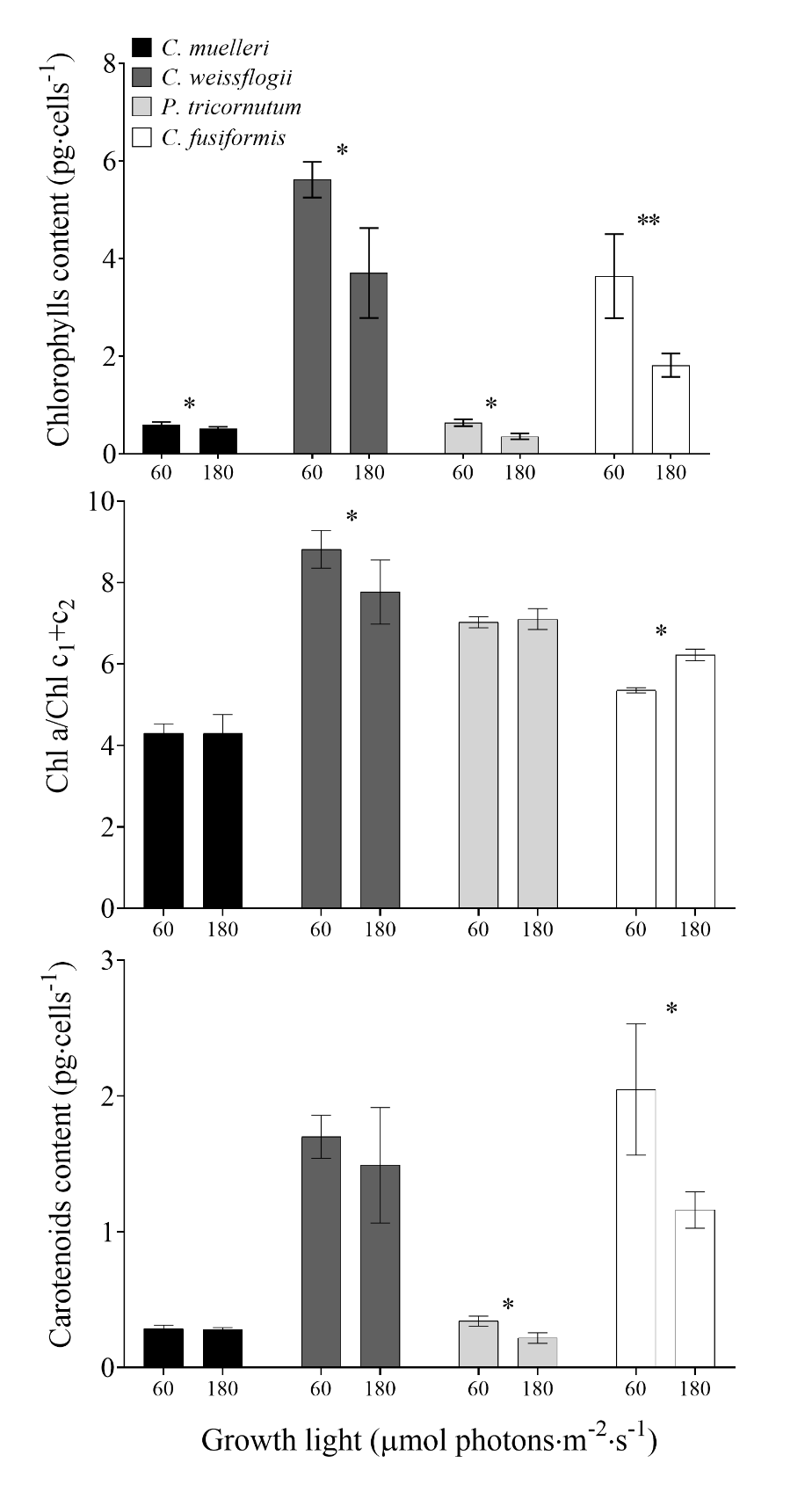


A

B

C

Supplementary Figure 2. Pigments analysis of the four diatoms acclimated to different light intensities: (A) chlorophylls quantification, (B) Chl*_a_*/Chl*_c1+c2_* ratio and (C) carotenoids quantification. Data are means of 3 biological replicas. Error bars show SD. Asterisks represent significant differences among conditions in the same species (* *p*<0.05, ** *p*< 0.001). Data were obtained as described in Petrucciani et al., 2022b.


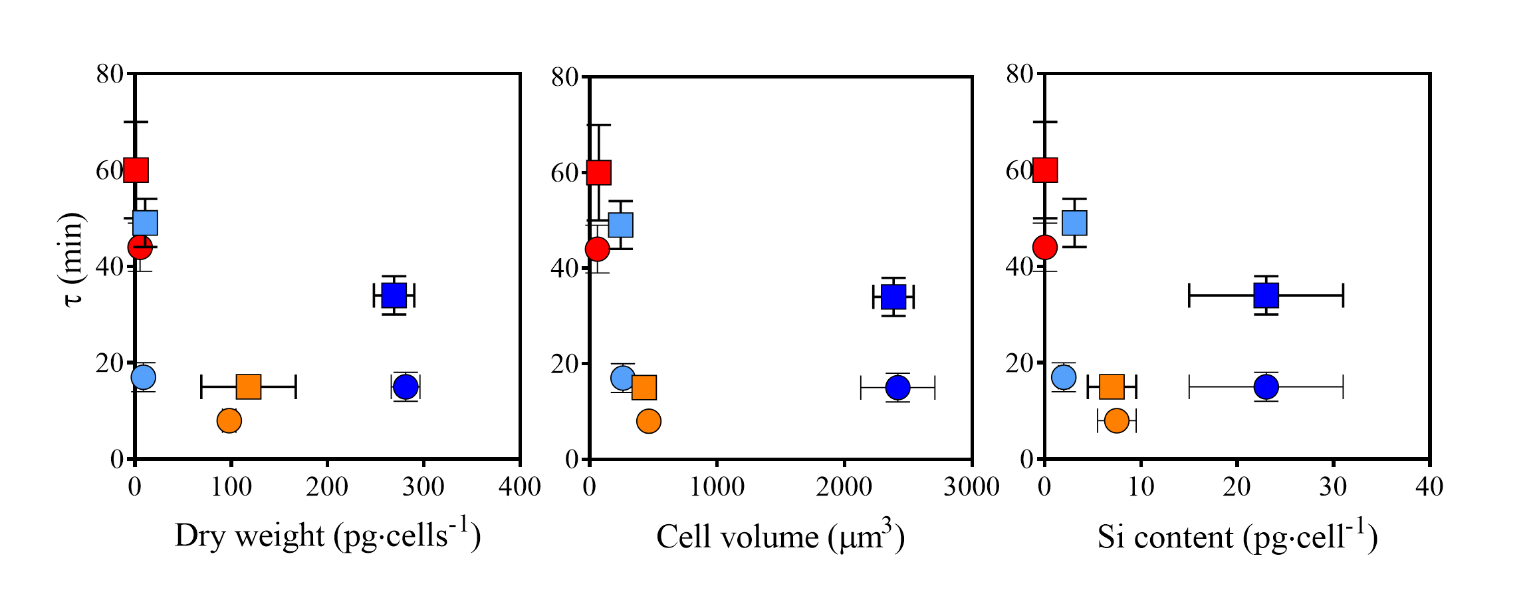


Supplementary Figure 3. τ values related to the dry weight, cell volume and Si content per cell in the four diatoms acclimated to 60 µmol photons·m^-2^s^-1^ (circles) and 180 µmol photons·m^-2^s^-1^ (squares). Centric species are shown by light blue (*C. muelleri*) and blue (*C. weissflogii*) symbols while pennate species by red (*P. tricornutum*) and orange (*C. fusiformis*) symbols. Error bars showed SD (when not evidenced, error bars are inside experimental points size).

Table S1. Mean ± SD of Cell volume (μm^3^) and Dry weight (pg) in the four diatoms acclimated to different light intensities (n ≥ 3). Asterisks indicate significant difference between conditions in the same species (*p* > 0.05).

|  |  | Growth Light (μmol photons⋅m^-2^⋅s^-1^) | |
| --- | --- | --- | --- |
| Cell volume (µm^3^) |  | 60 | 180 |
|  | *C. muelleri* | 262 ± 11 | 244 ± 23 |
|  | *C. weissflogii* | 2418 ± 290 | 2384 ± 158 |
|  | *P. tricornutum* | 60.67 ± 5.87 | 72.33 ± 10.97 |
|  | *C. fusiformis* | 464 ± 26 | 428 ± 43 |
| Dry weight (pg) | *C. muelleri* | 8.77 ± 0.88 | 10.63 ± 1.64 |
|  | *C. weissflogii* | 281 ± 15 | 269 ± 21 |
|  | *P. tricornutum** | 5.49 ± 0.92 | 1.14 ± 1.08 |
|  | *C. fusiformis* | 98 ± 7 | 118 ± 49 |

**Table S2**. K content (pg·cell^-1^) in the four diatoms acclimated to different light intensities. Data are means of 3 biological replicas ± SD.

|  |  | Growth Light (μmol photons⋅m^-2^⋅s^-1^) | |
| --- | --- | --- | --- |
|  |  | 60 | 180 |
| K content (pg·cell^-1^) | *C. muelleri* | 0.12 ± 0.02 | 0.21 ± 0.11 |
|  | *C. weissflogii* | 0.14 ± 0.04 | 0.17 ± 0.04 |
|  | *P. tricornutum* | 0.00200 ± 0.00001 | 0.002 ± 0.001 |
|  | *C. fusiformis* | 0.03 ± 0.01 | 0.030 ± 0.006 |

**Table S3.** Sedimentation rates (all expressed in μm·s^-1^) calculated from comparison with *P. tricornutum* estimated rate reported in Hamano et al., 2021. The velocity value $v_{PTL}$ reported in bold in the table, assimilated to our *P. tricornutum* grown at 60 μmol photons⋅m^-2^⋅s^-1^, is obtained by Hamano et al., 2021, and reported without standard deviation. Considering the corresponding sedimentation coefficient we obtained in the assimilated sample, $\tau_{PTL}=44 min$, we defined the equation $v_{i}=\frac{k}{\tau_{i}}$ where $k\cong8000\mu m$ as obtained from the product $v_{PTL}\cdot\tau_{PTL}=8000\mu m$. Consequently, for each experimental condition, $v_{i}$ values were estimated from the resulting $\tau_{i}$values reported in **Figure 1** of the manuscript without standard deviation.

|  |  | Growth Light (μmol photons⋅m^-2^⋅s^-1^) | |
| --- | --- | --- | --- |
|  |  | 60 | 180 |
| Estimated sedimentation rates (μm·s^-1^) | *C. muelleri* | 8 | 2.7 |
|  | *C. weissflogii* | 9 | 3.9 |
|  | *P. tricornutum* | **3** | 2.2 |
|  | *C. fusiformis* | 17 | 9 |
